# The Effect of Group Size on the Interplay between Dominance and Reproduction in *Bombus terrestris*

**DOI:** 10.1371/journal.pone.0018238

**Published:** 2011-03-28

**Authors:** Etya Amsalem, Abraham Hefetz

**Affiliations:** Department of Zoology, George S. Wise Faculty of Life Sciences, Tel Aviv University, Ramat Aviv, Israel; Georgia State University, United States of America

## Abstract

Social insects provide good model systems for testing trade-offs in decision-making because of their marked reproductive skew and the dilemma workers face when to reproduce. Attaining reproductive skew requires energy investment in aggression or fertility signaling, creating a trade-off between reproduction and dominance. This may be density-dependent because the cost of achieving dominance may be higher in larger groups. We investigated the effect of group-size in *B. terrestris* queenless workers on two major reproduction-dominance correlates: between-worker aggression, and pheromone production, aiming at mimicking decision-making during the transition of worker behavior from cooperation and sterility to aggressive reproductive competition in whole colonies. Despite the competition, reproductive division of labor in colonies can be maintained even during this phase through the production of a sterility signal by sterile workers that has an appeasement effect on dominant nestmates. Worker-worker aggression, ovary activation, and production of sterility-appeasement signals may therefore constitute components of a trade-off affecting worker reproduction decisions. By constructing queenless groups of different size and measuring how this affected the parameters above, we found that in all groups aggression was not evenly distributed with the α-worker performing most of the aggressive acts. Moreover, aggression by the α-worker increased proportionally with group-size. However, while in small groups the α-worker monopolized reproduction, in larger groups several workers shared reproduction, creating two worker groups: reproductives and helpers. It appears that despite the increase of aggression, this was evidently not sufficient for the α-worker to monopolize reproduction. If we compare the α-worker to the queen in full-sized colonies it can be hypothesized that worker reproduction in *B. terrestris* colonies starts due to a gradual increase in the worker population and the queen's inability to physically inhibit worker oviposition. This may shift the trade-off between cost and benefit of worker reproduction and trigger the competition phase.

## Introduction

The balance between cost and benefit is a powerful driving force underlying decision-making in animal societies. Since individuals are selected to maximize their fitness, they are predicted to evolve a decision-making process that will be the most adaptive under given ecological and socio-biological constraints. Social insects provide good model systems for testing trade-offs in decision-making because of their marked reproductive skew, constantly facing the worker caste with the dilemma of whether and when to reproduce [1]. Trade-offs in reproduction may involve many aspects of behavior. Among other factors, attaining reproductive skew in favor of one female requires her to invest energy either in aggression or in chemically signaling her fertility, thus creating a trade-off between reproduction and dominance [2]. This trade off may be quantity or density dependent since the cost of achieving dominance in larger groups is not equivalent to that in smaller groups, therefore, it is not surprising that group-size has a profound effect on the behavior and physiology of the individuals that compose it, especially among groups that cooperate together such as social insects. Since social insect colonies tend to progressively comprise increasing numbers of individuals, in cases where nest size is volume-limited (such as in the cavity-nesting *B. terrestris*) [3], the density effect (i.e. the number of individuals per unit area) cannot be disentangled from the group-size effect (i.e. the number of individuals). We will, therefore, use the phrase “quantity/density effect” to describe the effect of both, while using the term “group size” as to describe the factual quantity effect.

Differences associated with group-size may explain the complexity of whole colonies as well as the variation between individuals within the same species. In social insects, for instance, colony size effect has been suggested to be intimately related to kin structure [4], jointly providing a holistic explanation for colony complexity, namely, the suite of traits that together define the level of sociality. For example, worker polymorphism, morphological differences between castes, reproductive division of labor and complex communication systems are generally considered to be associated with large colony size [5]. Conversely, small colony size has been associated with higher reproductive potential of workers on the one hand and greater ability of the queen to suppress worker reproduction on the other hand, leading to intense and direct conflict over reproduction. In annual societies in which potential conflicts over reproduction may be time-constrained, inter-individual differences may be further accentuated.

Several studies have attempted to explore inter-individual differences on phenomena such as reproductive skew, aggression and dominance, but these have mainly considered group-level effects. For example, using a mathematical model, Cant and English [6] predicted a positive correlation between group-size and the proportion of females with fully-developed eggs, for which they presented evidence in the case of *Polistes dominulus*. Similar results were obtained in queenless workers of *B. impatiens* [7]. These cases suggest a link between group-size and either worker ovarian development, or the probability of an individual to reproduce.

Aggression is the clearest manifestation of reproductive conflicts and is believed to be a parameter for testing reproductive skew theory [8]. Several models have produced testable predictions regarding the correlation of aggression with group-size, with all reaching the same conclusion that aggressive behavior should be higher in larger groups [8]–[10]. However, to date, none were able to show this experimentally [8], [11]. Another, indirect facet of aggression is the establishment of a dominance hierarchy which is used by individuals to solve/avoid conflicts in a less costly manner. The evidence for group effect in this case is less controversial since both models and empirical data have revealed that both the number of individuals participating in the hierarchical ladder and the number of aggressive individuals increase with group (or colony) size [9]–[10], [12].

This study examined the effect of group-size on aggression and reproductive competition among *B. terrestris* queenless workers. Sociality in the bumblebee *B. terrestris* is typified by social phase transformation from harmonious cooperation among workers and absolute queen reproductive dominance to a highly aggressive competition between workers and the queen and among workers over male production during the so called competition phase [13]–[17]. However, not all workers in a colony have reproductive abilities, creating at least three social groups in the following order of dominance, the queen, fertile workers and sterile workers. The applicability of using queenless worker groups in our study is two folds. First, queenless workers are able to form hierarchy and division of labor as do fertile workers at the competition phase during the natural colony life cycle, which may shed light on some of the issues concerning the effect of worker quantity/density in full-sized colonies. Second, they mimic the situation of reproductive competition among equally apt individuals, thus serve for testing reproductive skew model and provide an insight into the relation between aggression and reproduction as part of the reproductive skew theory.

Earlier studies, including on dominance behavior [18], ovarian development [14], juvenile hormone titers and biosynthesis [19], ecdysteroids levels and aggressive behavior [20] and brain biogenic amines [21] were usually performed using 3-worker groups. These constitute the basis for our current understanding of worker behavior in queenless groups, as compared to queenright workers in a full-sized colony. In all of these, social conditions and density/quantity effects may have been confounded. A direct examination of the influence of group-size on workers in *B. terrestris* may disentangle these two factors.

Nests of *B. terrestris*, being founded by haplometrosis (a single queen), experience a gradual increase in worker population that reaches about 400 workers at the peak of colony development. Consequently, newly emerged workers face varying worker densities (ranging from 1 to 400 workers!) and thus may behave in a quantity/density-dependent manner. Despite the wealth of studies pertaining to the factors influencing the onset of the queen-worker competition phase, the actual trigger leading to this event is not yet fully understood, other than its clear correlation with the onset of gyne production [22]. Many of the hypotheses put forward, including the effect of worker quantity/density, were refuted or were not satisfactorily proven experimentally. Since the initiation of aggression and the transition of workers from sterility to reproduction in queenright colonies are accompanied by a gradual increase in group-size, it can be hypothesized that as worker quantity/density increases the queen loses control and workers tend to be more aggressive and eventually start ovipositing, which seems to shed further light on the trigger leading to competition in whole colonies.

Early work on social organization in animals assumed that a hierarchy was simply a culmination of dyadic relationships, the outcome of which was based only on factors intrinsic to the individuals such as size, age and physical prowess [23]. We have tried to minimize these individual factors by creating groups consisting of a-priori equal individuals. All were callow bees of comparable sizes, and were assembled from several mother colonies to ensure genetic variability. We then investigated the effect of group-size on aggressive behavior, reproduction, and the production of the postulated sterility signal in the Dufour's gland [24]. The quantity of this signal in subordinate workers is negatively correlated with ovarian development and aggression inflicted by the dominant bee [25].

## Materials and Methods

Colonies of *B. terrestris* were obtained from the Yad Mordechai Apiary, Israel; 3–5 days after the first worker had emerged. They were maintained in the laboratory in nest boxes (23×23×10 cm) at a constant temperature of 30°C and 50%–60% humidity, and supplied *ad libitum* with a sugar solution and fresh pollen collected from honeybee colonies. Callow workers (less than 24 h old) were removed from their mother colonies (n = 10 colonies), individually marked and kept for five days in queenless groups consisting of 3, 5 or 10 callow workers each (n = 12 groups for each group-size) Since callow workers were shown to behave as a “clean slate” [25], grouping was done randomly to eliminate genetic bias. Bees of each group were approximately of the same size, which was verified at the end of the experiments by measuring the width of the head capsule between the two compound eyes. No significant differences were found in the average worker size across group size categories (one-way ANOVA f_2,33_ = 1.78, p = 0.18 followed by Tukey-type post-hoc test, p>0.05). In addition, the variances in body size of each group were found homogeneous (Levenes' test for equal variances: p = 1 for 10-worker groups, p = 0.079 for 5-worker groups, p = 0.985 for 3-worker groups). The workers were placed in wooden nest boxes, the size of which was proportional to the group-size, so as to provide equal nest space per bee irrespective of group-size. Workers were supplied with the same diet of sugar solution and fresh pollen *ad libitum* throughout the experiments. Observation protocol was as follows: On the first day, the bees were observed only once, 5–8 hours post group establishment, after ascertaining that they had acclimated to the new conditions. During days 2–4 each group was observed for 10 minutes, 3 times a day, at fixed hours (10:00, 14:00 and 18:00). On the fifth day the bees were observed only twice, at 10:00 and 14:00, and were then sacrificed. The following behaviors were registered [25]–[26]: (a) “Attack”: occurrence of one of the following behaviors: biting, pushing, dragging, wing pulling, struggling or an attempt to sting. (b) “Darting”: a bee makes a sudden movement forwards in the direction of another bee, but without body contact between the two. (c) “Humming”: a series of wing vibrations lasting less than 3 seconds, performed by workers while they are active. For the humming behavior it was impossible to determine directionality. An index of aggression was constructed as the unweighted sum of “Attack”, “Darting” and “Humming” that each bee performed during all the observations throughout the entire experiment (12 observations for each group). Using this index we categorized workers in each group from the most dominant (α-female) to the least dominant.

At the end of the experiment, the bees were sacrificed by freezing and kept at −20°C until dissection. Dissections were done under a stereo-microscope in double-distilled water. The length of the terminal oocyte in the three largest ovarioles (at least 1 ovariole per ovary; workers possess 4 ovarioles per ovary) was measured with a scaled ocular. Mean terminal oocyte length for each bee was used as an index of ovarian development [27]. During the dissection, Dufour's glands were cleanly separated from the sting apparatus and extracted in 50 µl pentane containing 1 µg eicosane as internal standard. The samples were kept at −20°C until analysis.

Chemical analyses were performed by gas chromatography using DB-1 fused silica capillary column (30 m×0.25 mm ID) under a temperature program from 170°C to 300°C at 4°C/min. Compound identity was ascertained by GC/MS and retention times as compared to synthetic compounds [24]. Compound quantification was achieved by GC peak integration compared to the internal standard under the same chromatographic conditions. Data on ester distributions in entire colonies under three social conditions (queenright colonies before and at the competition phase and queenless colonies) were taken from a previously published study [24].

Statistics: statistical analyses were performed using Statistica for Windows, version 8.0. Comparisons of the average aggression and ovarian development were done using one-way-ANOVA followed by Tukey-type post-hoc test. The same analysis was used to test the fraction of aggression by the α-female. Proportions were transformed using arc*sin (p^∧^0.5) transformation before the parametric analyses. Comparisons of aggression, ovarian development and the amount of esters in the Dufour's gland in α-worker and β-workers under the three treatment groups were tested using two-way ANOVA followed by Tukey-type post-hoc test. The percentage of aggression exhibited by the α-worker towards the other females in a group was compared to percentage of aggression of the α-worker towards randomly assigned workers using χ^2^ test (observed vs. expected frequencies). Comparisons of variances for body-size was done using Levene's test of homogeneity of variances using QI Macros 2010. Statistical significance was accepted at α = 0.05. Data are presented as means ± SE.

## Results

### Average aggression and ovarian development per group

Considering all the workers in the group, group-size affected neither the average aggressive behavior nor the degree of ovarian development. Levels of aggression were 26.2±11.4, 32.5±14.5 and 33±16.2 aggressive behaviors in 120 min for groups of 3, 5 and 10 workers, respectively (one-way ANOVA f_2,33_ = 0.54, p = 0.58 followed by Tukey-type post-hoc test p>0.05). Regarding ovarian development (mean 3 largest oocytes), there were significant differences among groups: 0.44±0.09, 0.35±0.08 and 0.52±0.1 mm for groups of 3, 5 and 10 workers, respectively (one-way ANOVA f_2,33_ = 3.43, p = 0.04 followed by Tukey-type post-hoc test p = 0.03 for 10-worker groups vs. 5-worker groups and p>0.05 for the rest). However, neither aggression nor ovarian developments were evenly distributed among the bees of each group.

### Aggression in α- and β-workers

**Figure 1:**
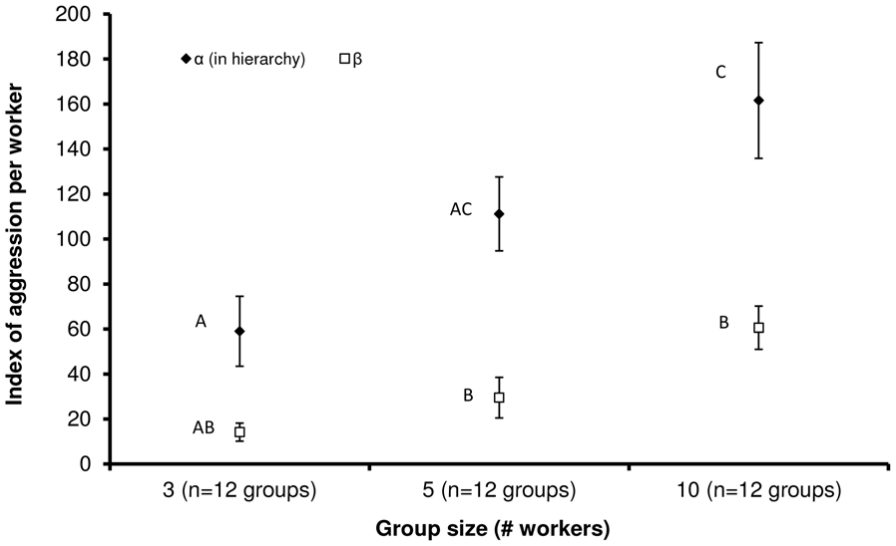
Aggression level per worker in α-workers and β-workers in 5-day-old workers. Workers were kept in queenless groups of 3, 5 and 10 (12 groups for each group-size). Each group was observed for a total of 120 minutes. Data are presented as mean ± SE for the α-workers and β-workers in each group. Different letters denote statistical differences using two-way ANOVA test.

The α-worker performed most of the aggressive acts, irrespective of group-size, and her cumulative aggression was group-size dependent. Figure 1 presents the effects of group-size on the level of aggression exhibited by the α- and β-females. Both “worker's position in the hierarchy” and “group-size” had a significant effect on aggression levels in workers, but there was no interaction between the two factors (two-way ANOVA for group-size: f_2,66_ = 11.69, p<0.001; for place in hierarchy: f_1,66_ = 34.4, p<0.001; for group-size*place in hierarchy: f_2,66_ = 1.36, p = 0.26). For both the α- and β-females the observed aggression level increased in correlation with the size of the group (Tukey-type post-hoc test p<0.05 for groups of 10 workers vs. groups of 3 and 5 workers). In addition, the α-females showed a significantly higher aggression index compared to β-females in two out of three tested groups (Tukey-type post-hoc test p<0.01 for α-females vs. β-females in groups of 5 and 10 workers). Among the α-females, those that were in groups of 10 were significantly more aggressive than the α-females in groups of 3 (Tukey-type post-hoc test p<0.001). A similar tendency for augmented aggression in large groups was also noted for the β-females, but the differences were not statistically different (Tukey-type post-hoc test p>0.05).

**Figure 2:**
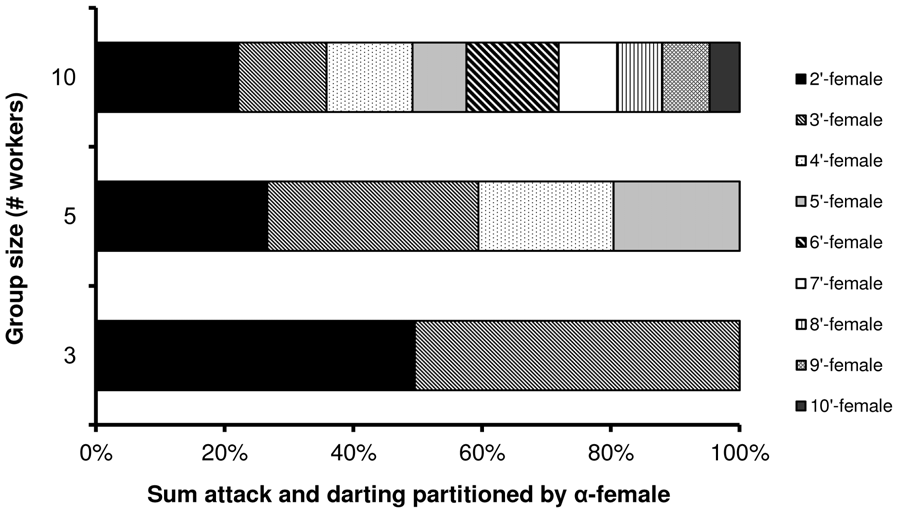
Percentage of aggression exhibited by the α-worker towards the other females in the group. Aggression level includes cumulative aggression (attack and darting) during 5 days. Humming was excluded because of inability to determine directionality with certainty. Each group was observed for 120 minutes. Workers were kept in queenless groups of 3, 5 and 10 (12 groups for each group-size). Workers are presented in accordance with the amount of aggression each has received from the α-worker (e.g. 2′ female received the highest amount of aggression).

Regardless of group-size, most of the aggression within a group was performed by the α-female. The percentage of darting and attacks that were made by the α-females (84±9%, 73±13% and 95±4% attacks in groups of 3, 5 and 10, respectively, and 77±8%, 59±11% and 71±9 darting in groups of 3, 5 and 10, respectively) were similar in all examined groups (one-way ANOVA for attacks: f_2,33_ = 0.78, p = 0.46, for darting: f_2,33_ = 0.25, p = 0.77). In contrast, the percentage of humming by the α-females in 10-worker groups was lower than that by the α-females in 3-worker groups (69±6%, 63±6% and 47±4% humming in groups of 3,5 and 10, respectively) (one-way ANOVA f_2,33_ = 5.38, p = 0.009 followed by Tukey-type post-hoc test p = 0.008). Figure 2 presents the partitioning of aggression made by the α-worker towards each of her group mates. Since the directionality of humming could not be determined, and the cumulative HDT (humming + darting + attacks) in Figure 1 was also comparable to those of the cumulative DT (darting + attack), humming was omitted from the figure. While in groups of 3 and 5 workers, the null hypothesis that aggression was equally directed towards all group mates was accepted, in the groups of 10 workers aggression was differentially directed, with the β-workers receiving about two-fold aggression (22.1% on average) than that expected at random (11.1%), while the lowest female in the hierarchy receiving about half the aggression of that expected at random (4.68% on average) (observed vs. expected frequencies: χ^2^ = 20.41, df = 8, p = 0.008 for groups of 10, χ^2^ = 4.32, df = 3, p = 0.22 for groups of 5 and χ^2^ = 0.09, df = 1, p = 0.92 for groups of 3). Despite the clear bias in aggression partitioning made by the α-worker towards the bee next in hierarchy in the 10-bee groups, the β-worker (and to some extent also the γ-female) still received less aggression compared to those in the smaller groups.

### Ovarian development in α- and β-workers

Worker ovarian development was affected both by “group-size” and “worker position in the hierarchy” (Table 1; two-way ANOVA for group-size: f_2,66_ = 5.27, p = 0.007 for; for position in hierarchy: f_1,66_ = 5.92, p = 0.017; for group-size*position in hierarchy: f_2,66_ = 2.53, p = 0.087). The α-workers in each group-size revealed the greatest ovarian development, and there was no difference among the α-workers of the different groups (Tukey-type post-hoc test, p>0.05). Notwithstanding, the β-females of the 10-worker groups had significantly larger oocyte development compared to that in the 3-worker groups (Tukey-type post-hoc test, p<0.05). The β-workers in the 5-worker groups had intermediate ovarian development that did not differ either from that in the 10-bee groups or that in the 3-bee groups (Table 1). For comparison purposes we set the lower threshold for developed ovaries at an oocyte size greater than 0.5 mm, since the workers were sacrificed before full ovarian development was achieved. While in groups of 3 and 5 workers only 1.08±0.22 and 1.25±0.3 workers, respectively, fitted this criterion (n = 12 for each size group), there were 4.5±0.77 such workers in groups of 10 (n = 12; Table 1).

**Table 1:** Ovarian development and amount of esters in Dufour's gland of α- and β-workers in 3 different group-sizes.

Bee category	Group-size		
	3 (n = 12)	5 (n = 12)	10 (n = 12)
Ovarian development-mm of largest oocyte (mean±se)			
α-workers	0.77±0.1^A^	0.53±0.1^AB^	0.89±0.15^A^
β-workers	0.31±0.05^B^	0.52±0.09^AB^	0.75±0.1^A^
No. of bees with developed ovaries	1.08±0.22	1.25±0.3	4.5±0.77
Ester amounts (µg) (mean±se)			
α-workers	0.8±0.27^a^	0.88±0.28^a^	0.24±0.07^a^
β-workers	3.48±1.33^b^	1.18±0.44^ab^	0.23±0.08^a^

The experiments were performed in 5-day-old workers that were kept in queenless groups of 3, 5 and 10 (12 groups for each group-size). Data are presented as mean ± SE for the α-workers and β-workers in each group. Different letters denote statistical differences using two-way ANOVA test.

### Dufour's gland secretion

**Figure 3:**
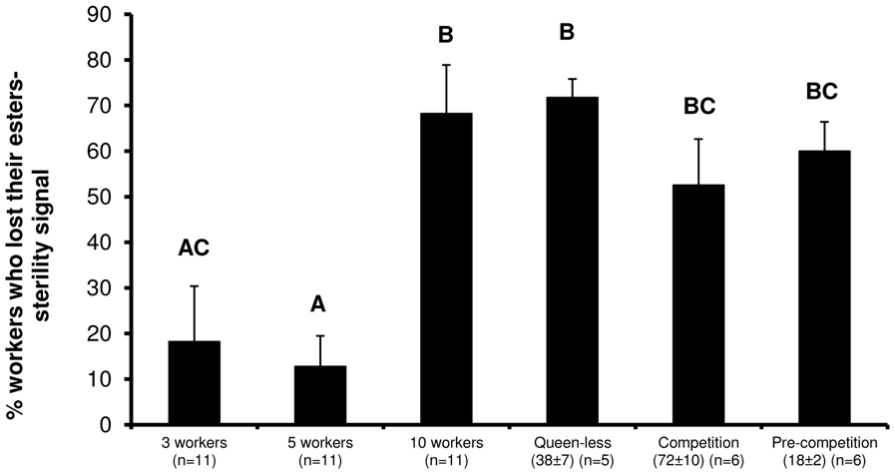
The percentage of workers that lost their Dufour's gland esters in queenless groups and colonies. Workers with less than 1% esters per total secretion were defined as workers that had lost their ester-sterility signal. The workers were 5-day-old and were kept in queenless groups of 3, 5 and 10. Workers' age in colonies was distributed normally (for more details see [24]). The numbers in brackets denote the numbers of workers per colony and the size of the sample (number of groups or colonies). All the workers in each group were dissected, therefore the n describes the number of Dufour glands as well. Letters above the columns denote statistical differences at p<0.05. Data are presented as mean ± SE.

The average amounts of esters in Dufour's gland in α- and β-females are presented in Table 1. Both “worker position in the hierarchy” and “group-size” had significant effect on the amount of esters in Dufour's glands of workers, but no interaction was found between the two parameters (two-way ANOVA for group-size: f_2,65_ = 4.93, p = 0.01; for position in hierarchy: f_1,65_ = 3.99, p = 0.049; for group-size*position in hierarchy: f_2,65_ = 2.88, p = 0.063). Considering all the bees in the group, the average amounts of esters decreased in correlation with group-size (Tukey-type post-hoc test, p = 0.005 for groups of 10 workers vs. groups of 3 workers). Examination of ester amounts in the α-females and β-females revealed that for the α-females the average amounts were very low and did not differ between the three treatments (Tukey-type post-hoc test p>0.05). For the β-females, on the other hand, this decreased in correlation with the size of the group (Tukey-type post-hoc test p = 0.004 for β-females in groups of 3 vs. β-females in groups of 10). Consequently, there was a significant difference is ester amounts between the α- and β-females in groups of 3 workers (Tukey-type post-hoc test p = 0.048). Figure 3 presents the percentage of workers that lost their esters, i.e. had less than 1% esters per total secretion in their Dufour gland, in the experimental worker groups as well as in entire colonies. The percentage of workers that lost their esters in small queenless groups (3 and 5 workers) was significantly different from that in both 10-worker groups and full-sized colonies, whether queenless or queenright before or at the competition phase (one-way ANOVA: f_5,44_ = 6.64, p<0.001 followed by Tukey-type post-hoc test, p<0.05). There were no significant differences in the percentage of workers without esters in the 10-worker groups compared to full-sized colonies, despite the fact that the former were 5-day-old whereas worker age in the colonies was normally distributed (for details and statistics see reference [24]).

## Discussion

The results obtained in the present study demonstrate that reproductive competition among queenless *B. terrestris* workers develops along similar pathways as in whole colonies during the competition phase. It also revealed similarities with the behaviors leading to reproductive skew among cooperating individuals. Aggression has long been postulated to be a key parameter when modeling reproductive skew. According to most of these models, although aggression by the subordinate is predicted to positively correlate with reproductive skew [8], [28]–[30], several experimental results in primitively eusocial wasps and in the current study refuted this prediction [31]–[33]. These differences may stem from the fact that hierarchy in some eusocial insects is dynamic rather than fixed. In such cases, a subordinate with the same potential to compete over reproduction, will tend to increase aggression in order to attain the dominant position rather than merely achieve equal reproduction portion ( = low reproductive skew). This aggressive tendency may be especially reinforced in societies which are seasonally constraints, as in the case with *B. terrestris*. In our experimental groups, aggression levels expressed by individuals at the top of the hierarchy (in particular by the α-female, but also by the β-female) become more intense with increase in group-size. Importantly, the α-workers in groups of 3 were able to attain absolute reproductive skew using relatively low aggression, whereas in the 10-worker groups they could not reach such a degree of absoluteness despite exhibiting much greater aggression. This suggests that reproduction in the lower-ranked bees stems either from the inability of the α-worker to repress their ovarian development or from the α-worker restricting her aggression in order to maintain group integrity (as assumed by the reproductive skew models [29]). We find the first option more likely since there is no point in α-worker restricting her aggression towards competing workers as long as there are enough submissive workers that can support full group productivity, as we observed in the 10-worker groups. Furthermore, results obtained by several studies implied that because of their annual life cycle and the onset of the competition towards the end of colony life cycle, competition between *B. terrestris* workers culminates on who is the first to lay eggs, as well as laying the maximal egg number possible. The first worker to lay eggs will have the highest chance to successfully rear her males and synchronize their emergence with the gynes' emergence, thus also insuring the male's mating success [22], [34]–[35]. In view of the above we conclude that exerting absolute dominance is crucial and therefore, reproduction self-restriction by the α-female or reproductive sharing because it is ineffective to lay eggs above of the group capacity, are unlikely to occur.

Another important point stemming from our results is that when correlating aggression with group-size, calculating aggression-averages including all bees in the group may be misleading. While in our study with *B. terrestris* the average levels of aggression showed no group-size effect, it was clear that aggression was not evenly distributed among group members, with the α-worker performing over 70% of the aggressive acts. Thus, if we consider the α-worker alone, there is a positive relationship between group-size and the magnitude of aggression. Our results are in line with the model presented by Cant et al. [8] but not with their results.

The pattern of Dufour's gland sterility signal production among workers sheds further light on the effect of group-size. While in small groups containing 3 and 5 workers, only one worker on average (the α-female) had lost her esters, about 60% of the workers in groups of 10 had lost their esters. This percentage is similar to that found in full-sized QR colonies, before or at the competition phase [24], as well as in queenless colonies (Figure 3), suggesting that groups of 10 workers, but not of 3 or 5 workers, may best represent the pheromonal and reproductive status of workers in full-sized colonies.

We further demonstrated that the β-females in the larger groups lose their esters earlier than the β-females in smaller groups (ester loss in the α-females was equally rapid irrespective of group size). This finding matches the higher ovarian development of β-workers in the larger groups and strengthens the correlation between aggressive behavior and ester disappearance, as described previously [24]–[25]. It also implies that β-workers in larger groups, as in whole colonies, advertize to the α-female (worker or queen) as well as to other workers, that they are no longer sterile workers that maintain the harmony, but fertile individuals entering the circle of reproduction and fighting for their dominance placement within the group (colony). We thus suggest that the 10-worker groups already shows a reproductive division of labor in which about 40% of the workers have no chance of reproducing and thus focus their efforts on brood care. The advertizing of sterility through ester production by these workers [24] helps to maintain the harmony within the nest, presumably increasing total group reproductive output.

Although in our experiments we used queenless worker groups, we suggest that this can explain many of the queen-worker interactions in full-sized colonies. We suggest equaling the α-worker and β-worker in the QL groups to the queen and potentially reproductive elite-worker [36] in a QR colony before the competition starts, respectively. Such comparison is sensible for several reasons: the queen in *B. terrestris* is always the α-female [15], [18], [36]–[38]; the establishment of a dominance hierarchy in queenless groups is very similar to that observed when bumblebee queens are confined together shortly after termination of hibernation in commercial hives [39]. In both cases the dominance hierarchy is established using overt aggression and maintained by dominant-subordinate interactions in which the dominant starts ovipositing and the subordinate rarely lay eggs [18]; both the queen and the α-worker are able to inhibit egg formation in subordinates [17], [37]; and finally, although fertile workers cannot mate, they still possess a spermatheca. They also have the same number of overioles as queens and they mimic the queen's Dufour gland composition in lacking the ester-sterility signals, unlike sterile workers who possesses extra compounds [24]. It is therefore reasonable to assume that the mechanisms underlying reproduction dominance in queens and fertile workers are similar. Colonies of *B. terrestris* present a transition from within-colony harmony to aggressive competition. Although *B. terrestris* colonies may contain hundreds of workers, the competition point may be reached while the colony contains only few dozens of workers [13]. Moreover, the competition starts with no delay even when limiting the number of workers to 20 [41]. Although this transition occurs according to a predictable timetable, the trigger for its onset remains elusive. Several hypotheses have been suggested, including (1) the timing of the switch point (the switch made by the queen from laying diploid to haploid eggs [38] (see [13], [42]–[44] for criticism); (2) the decline in queen aggression, fertility, or ability to inhibit worker reproduction [13], [15]–[16], [36], [45]; and (3) the ascertainment by the workers that larvae are committed to gyne development [22], [40], [44] (see [13], [17] for criticism).

Most of the empirical evidence in support of each of the above hypotheses remains controversial. A fourth hypothesis was raised already in 1949 by Cumber, who suggested that egg-laying by workers depends on their number or density in the brood area [15], [36], [46]–[48]. Although many other authors have discussed this question, only one study has experimentally supported the hypothesis [17] while others have criticized it, mostly providing indirect evidence [13], [41], [49].

We suggest here a density effect, i.e. a threshold number of workers at the onset of the competition point. Below that threshold the worker with the most developed ovaries can be still inhibited by the queen (as were the β-workers in the small QL groups). Above this threshold, however, although the queen exercises all her potential power in inhibiting the workers from laying eggs, this is apparently insufficient to inhibit workers from egg-laying (as occurred in the 10-worker QL groups).

Our results are in line with earlier experiments in which manipulation of small-size colonies affected the onset of the competition point and gyne production [17]. However, they may seem inconsistent with [41], who did not find an effect on the competition point when worker number was limited to 20, and with [13], who reported the same competition point in lab-rearing colonies and free-foraging colonies, although the former was twice as large. We propose that since in both cases the number of workers exceeded 10, at which size, according to our results, workers behave the same as in a full-sized colony regarding ovarian development and pheromone production, we should not expect any differences.

Since some of the workers in full-sized colonies refrain from reproduction despite having fully developed ovaries already during the pre-competition phase, the transition from sterility to fertility cannot be explained by the quantity/density effect alone. We therefore suggest to couple between two explanations: at the proximate level, the queen behaviorally inhibits workers from laying eggs. This is in line also with the ultimate cause, since also workers have an incentive to defer their reproduction until (1) gyne development is underway, or/and (2) there is sufficient worker force to support reproductive production as well. Indeed, the latter constraint may explain why isolated workers delayed ovarian development even when away from the influence of the queen [24]. Workers gain more fitness from rearing gynes than from rearing sons, and assuming that the whole population behaves similarly, there is no point in rearing sons if this hampers gyne rearing. Since the queen has no other alternative than to rear gynes before the end of the season, workers will be selected to cooperate while waiting for the optimal group-size that provides the best chances for successful worker-derived male production.
